# AIMP-Based Power Allocation for Radar Network Tracking Under Countermeasures Environment

**DOI:** 10.3390/s25103163

**Published:** 2025-05-17

**Authors:** Xiaoyou Xing, Longxiao Xu, Lvwan Nie, Xueting Li

**Affiliations:** 1 School of Aeronautics and Astronautics, Sichuan University, Chengdu 610065, China; 2022141510062@stu.scu.edu.cn; 2 School of Electronic Engineering, University of Electronic Science and Technology of China, Chengdu 611731, China; 202111012117@std.uestc.edu.cn (L.X.); 202322010323@std.uestc.edu.cn (L.N.)

**Keywords:** power allocation, target RCS, pulse intercept, suppressive jamming

## Abstract

For radar system tracking, a higher radar echo signal to interference and noise ratio (SINR) implies a higher tracking accuracy. However, in a countermeasures environment, increasing the transmit power of a radar may not lead to a higher SINR due to suppressive jamming. Also, the variation in the target radar cross-section (RCS) is an important factor affecting the SINR, since to achieve the same SINR value, a large RCS value needs less transmit power and a small RCS value needs more transmit power. Therefore, to design an efficient power allocation strategy, the influence of the electronic jamming and the target RCS need to be jointly considered. In this paper, we propose an adaptive interacting multiple power (AIMP)-based power allocation algorithm for radar network tracking by jointly considering the electronic jamming and the target RCS, achieving better anti-jamming capability and lower probability of intercept (LPI) while not reducing the tracking accuracy. Firstly, the model of the radar network tracking is established, and the power allocation problem is formulated. Next, the target RCS prediction algorithm is introduced, and the AIMP power allocation method is proposed jointly considering the electronic jamming and the impact of the target RCS. Finally, numerical simulations are performed to verify the validity and effectiveness of the proposals in this paper.

## 1. Introduction

In modern airspace defense with excellent performance, e.g., anti-jamming, the radar network is playing an increasingly important role. Radar tracking is regarded as a relatively significant task in airspace defense, such that the target position can be continuously updated by radars for subsequent tasks, such as guidance. A high tracking accuracy is always pursued for radar networks, and the tracking performance depends on the signal to interference and noise ratio (SINR) of the radar echo signal at a large scale. According to the typical radar equation, the SINR of the radar echo signal is directly proportional to the radar transmit power and the target radar cross-section (RCS). Thus, an effective radar transmit power allocation strategy is an important approach to improving tracking accuracy. For example, two optimal resource (radar transmit power, dwell time, etc.) allocation schemes are proposed in [1,2], achieving maximal multi-target tracking accuracy for a heterogeneous radar network with a given resource budget.

However, with the rapid development of electronic warfare technology [3,4], the complicated electromagnetic environment raises new challenges for power allocation in radar networks. In a countermeasures environment, the target is usually equipped with the interceptor, and the excessive transmit power may cause the pulse to be intercepted. Typically, the transmit signal of a radar is usually composed of multiple pulses within a tracking frame (hereafter referred to as frame), and once the intercepted pulse number accumulates to a certain threshold, the signal will be identified by the interceptor and the radar will be jammed [5,6], significantly affecting the SINR and tracking accuracy. Therefore, the problem of low probability of intercept (LPI) [7,8] has received attention in the power allocation problem for radar network tracking. For instance, with the parameters of the interceptor available, an optimization problem for joint power allocation and waveform selection strategy is formulated to improve the target tracking accuracy and LPI performance. A joint target assignment and resource optimization strategy for radar network tracking is proposed in [9] under resource constraints, and the strategy is demonstrated to be advantageous in improving tracking accuracy as well as LPI performance.

The aforementioned works have made contributions to improving tracking accuracy (the SINR) and LPI performance. As noted at the beginning of this paper, the target RCS is also an issue that is worth considering for improving the SINR of the radar echo signal. The target RCS is usually described by the average value as well as the probability density function family [10], such as Swerling I–IV models [11]. Researchers have paid attention to the target RCS for its effects on the power allocation strategy in a radar network. For example, the target RCS is assumed to follow a first-order Markovian process in [1,12,13], wherein the target RCS and state are extended as one vector and estimated together. Considering the probabilistic uncertainty on target RCS, a robust chance-constrained-based power allocation scheme is proposed in [14], improving power utilization efficiency.

In practical applications, the target will provide a rich scattering environment yielding 5–20 dB RCS fluctuations [15], when it is radiated from different directions by radars [16]. In other words, the target will exhibit different RCS values when radar radiates on its different **sectors**. Taking the aircraft for example, its RCS value is usually smaller when the radar radiates from the frontal sector, while it will be larger when the radar radiates from the lateral sector; thus, modern stealth fighters typically reduce the backscatter in the frontal area to reduce RCS [10].

**Figure 1:**
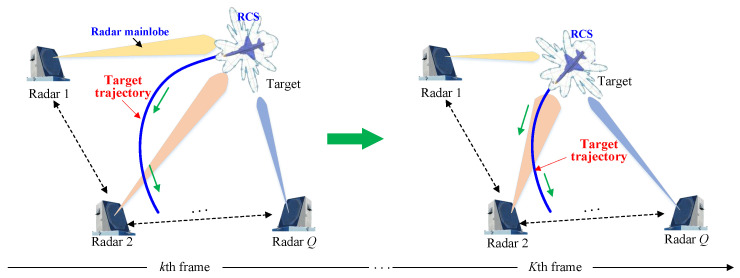
Schematic diagram of target RCS variation with target motion in the surveillance area of the radar network.

If the prior knowledge of RCS value distribution can be obtained with the help of military intelligence or the publicly available information, the target scattering area can be divided into different sectors, and thereby the RCS value according to the target sector that is radiated by the radar can be derived. Obviously, when the target motions in the monitoring area of the radar network are with long baselines, the target sectors that are radiated by each radar will be different, and the target RCS will exhibit spatial diversity. The process by which the target exhibits different RCS values to radars during the motion period is briefly depicted in Figure 1.

The sector on the target where the radars radiate from can be predicted according to the target trajectory, and thus, the target RCS exhibited to different radars can be predicted as well. This will be useful for guiding the adjustment of radar transmit power, especially in countermeasures environments, where the radars may be enabled to achieve equivalent tracking effects while consuming less power, further improving the LPI performance of the radar network.

However, under the constraints of a countermeasures environment where the radar network faces the threat of suppressive jamming, such as in our previous work [17], the power allocation algorithm only considers the target–radar range, and the target RCS is assumed to be a fixed value. To the best of the authors’ knowledge, the problem of adjusting the transmit power for radar network tracking jointly considering the target–radar range and the variation in target RCS is not yet available in the literature. This is indeed the main idea of this paper.

### 1.1. Main Contributions

The major contributions of this paper can be summed up as follows:

(1) *Build an optimization model of the power allocation for the radar network tracking in a countermeasures environment:* On one hand, if a radar is jammed by electronic suppressive jamming, the SINR of the radar will drastically decrease, which negatively affects the tracking accuracy. The target RCS can also affect the SINR, on the other hand. Therefore, the threat of suppressive jamming and the variation in target RCS during the target motion are both considered to build the power allocation optimization model for a radar network in tracking applications. Specifically, at a certain frame, the impact of the jamming on the echo pulses is regarded as the feedback, combined with the target RCS prediction at the next frame; these two factors jointly guide the transmit power adjustment of the radar network at the next frame.
(2) *Develop a target motion trajectory-based RCS prediction method:* In general, the target RCS depends on the sector where the radar radiates, and the sector can be described as the incidence angle with which the radar illuminates the target. With the target maneuvering, the incidence angle is decided by the trajectory within two frames of the target. The target state (i.e., the position) at the next frame is first predicted, and in turn, the geometric relationship between the target position within the two successive frames is obtained. The cosine theorem is then utilized to calculate the predicted radar incidence angle, and the target sector radiated by the radar can be determined. Combined with the sector-based RCS value distribution obtained from the prior knowledge, the predicted target RCS at the next frame can finally be obtained.
(3) *Propose an adaptive interacting multiple power (AIMP)-based power allocation algorithm for radar network tracking:* Predictably, in a countermeasures environment, there exists an optimal transmit power under a given target RCS that the radar network can achieve high SINR while maintaining better LPI performance. To achieve this strategy, based on our previous work, a power allocation strategy based on the IMP algorithm [17], combined with the prediction of target RCS to create an AIMP-based power allocation algorithm for the radar network, is proposed.

Some simulation results are given to show that, compared with the other algorithms without considering the target RCS, the radar network can achieve equivalent tracking performance by using less power with the proposed AIMP algorithm, while allowing the radar network to maintain better LPI performance.

### 1.2. Organization of This Paper

The rest of this paper is organized as follows: In Section 2, the tracking accuracy as well as the LPI performance are analyzed. The problem of power allocation for radar network tracking is formulated in Section 3. In Section 4, the RCS prediction method is introduced, and the AIMP-based power allocation algorithm for radar network tracking is proposed. Some simulation results are presented in Section 5. The conclusions are given in Section 6.

## 2. System Modeling

Considering a radar network composed of *Q* widely deployed monostatic radars continuously monitoring an airspace, the location of the *q*th (q=1,2,…,Q) radar is denoted as $\left(x_{q},y_{q}\right)$. Each radar in the network ensures time synchronization and can process only its own echo signals scattered by the target. Assume the presence of a mobile target equipped with an interceptor and countermeasure system (jammer) within the designated area. Subsequently, some key parts of this system model are presented.

### 2.1. Signal Model and SINR

Assume that radar *q* will transmit a constant of $N_{q,k}$ pulses during the *k*th frame, each pulse is assigned the equivalent transmit power, denoted as $P_{q,k}^{t}$. After reflection from the target, the *n*th echo pulse ($n=1,2,\ldots ,N_{q,k}$) at the *k*th frame of radar *q* can be expressed as

(1) $$\begin{array}{c} r_{q,n,k}\left(t\right)=\beta_{q,k}\sqrt{P_{q,k}^{t}\alpha_{q,k}}s\left(t\right)+J_{q,n,k}\left(t\right)+w_{q,n,k}\left(t\right) \end{array}$$

where $\beta_{q,k}$ denotes the target RCS with respect to radar *q*, s(t) represents the normalized complex envelope of the transmit signal, $\alpha_{q,k}$ is the variation in the signal strength due to the path loss. Additionally, $J_{q,n,k}\left(t\right)$ and $w_{q,n,k}\left(t\right)$ are suppressive jamming and Gaussian noise, which are denoted as zero-mean Gaussian processes with variance $\sigma_{ja}^{2}$ and $\sigma_{w}^{2}$, respectively. The SINR of the *n*th received pulse can be considered as a function of transmit power $P_{q,k,n}^{t}$, denoted by $\mu_{q,n,k}$ and represented by

(2) $$\begin{array}{c} \mu_{q,n,k}\left(P_{q,k}^{t},\beta_{q,k}\right)=\frac{P_{q,k}^{t}{\left(\beta_{q,k}\right)}^{2}\alpha_{q,k}}{\sigma_{w}^{2}+\sigma_{ja}^{2}}. \end{array}$$

Naturally, if radar *q* is not jammed, $J_{q,n,k}\left(t\right)=0$ and $\sigma_{ja}^{2}=0$ hold.

It appears that greater radar transmit power will yield a higher SINR when other variables are held constant. However, it is also worth mentioning that the variation in target RCS may allow more options for transmit power allocation. Specifically, less transmit power can be appropriate for the equivalent SINR requirement with a larger target RCS, and vice versa.

### 2.2. Target Motion Model and Radar Measurement Model

Consider a single target tracking scenario, and the state of the target is $X\left(k\right)={\left[x_{k},{\dot{x}}_{k},y_{k},{\dot{y}}_{k}\right]}^{T}$, where ${\left[\cdot \right]}^{T}$ is the transpose operator. $\left[x_{k},y_{k}\right]$ and $\left[{\dot{x}}_{k},{\dot{y}}_{k}\right]$ represent the position component and velocity component, respectively. Without loss of generality, the target state equation at the *k*th frame can be expressed as,

(3) $$\begin{array}{c} X\left(k\right)=F\left[k,X(k-1)\right]+\upsilon \left[k,X(k-1)\right] \end{array}$$

where $F\left[\cdot \right]$ denotes the state transition function, and $\upsilon \left[\cdot \right]$ denotes the process noise vector.

After undergoing the necessary signal detection steps, radar *q* might obtain $M_{q,k}$ measurements within the validation at the *k*th frame, denoted as ${Ƶ}_{q,k}=\left\{z_{q,k}^{1},z_{q,k}^{2},\ldots ,z_{q,k}^{M_{q,k}}\right\}$, and the *m*th measurement has a general form, expressed as

(4) $$\begin{array}{c} z_{q,k}^{m}=\left\{\begin{array}{cc} h\left[X(k)\right]+n_{q,k} & \;\mathrm{if}\;\mathrm{derived}\;\mathrm{by}\;\mathrm{target}\\ w_{k} & \mathrm{if}\;\mathrm{the}\;\mathrm{false}\;\mathrm{alarm} \end{array}\right. \end{array}$$

with

(5) $$\begin{array}{c} h\left[X\left(k\right)\right]={\left[R_{q,k},\theta_{q,k}\right]}^{T} \end{array}$$

where $h\left[\cdot \right]$ denotes the measurement function, and $R_{q,k}$ and $\theta_{q,k}$ are the range and bearing angle observed by the *q*th radar, calculated by

(6) $$\begin{array}{c} R_{q,k}=\sqrt{{\left(x_{q}-x_{k}\right)}^{2}+{\left(y_{q}-y_{k}\right)}^{2}} \end{array}$$

(7) $$\begin{array}{c} \theta_{q,k}=\arctan\frac{y_{q}-y_{k}}{x_{q}-x_{k}}. \end{array}$$

In addition, the term $n_{q,k}$ is the measurement error matrix with the covariance of $\Sigma_{q,k}$, given by

(8) $$\begin{array}{c} \Sigma_{q,k}=\mathrm{diag}\left(\sigma_{R_{q,k}}^{2},\sigma_{\theta_{q,k}}^{2}\right) \end{array}$$

where $\sigma_{R_{q,k}}^{2}$ and $\sigma_{\theta_{q,k}}^{2}$ represent the Cramér-Rao lower bounds (CRLBs) of the parameters $R_{q,k}$ and $\theta_{q,k}$, respectively [1,18]. These CRLBs can illustrate the estimation accuracy of the parameters and can be expressed as a function of SINR [13,19].

(9) $$\begin{array}{c} \left\{\begin{array}{c} \sigma_{R_{q,k}}^{2}\propto {\left[\mu_{q,k}{\left(B_{q}\right)}^{2}\right]}^{-1}\\ \sigma_{\theta_{q,k}}^{2}\propto {\left[\mu_{q,k}{\left(A_{q}\right)}^{2}\right]}^{-1} \end{array}\right. \end{array}$$

where $\mu_{q,k}=N_{q,k}\mu_{q,k,n}$ is the coherent accumulation SINR of radar *q* at the *k*th frame, $B_{q}$ is the signal effective bandwidth of radar *q*, $A_{q}$ is the antenna aperture, $w_{k}$ is the false alarm [2,20].

**Remark** **1.** *The smaller CRLBs imply higher estimation accuracy, and the SINR is inversely related to these CRLBs. In other words, allocating a more optimal transmit power can achieve a lower CRLB, i.e., higher tracking accuracy.*

The target state estimation can be summarized by the following equations [21,22],

(10) $$\begin{array}{c} \hat{X}\left(k|k-1\right)=\hat{F}\left[k,\hat{X}\left(k-1\right)\right] \end{array}$$

(11) $$\begin{array}{c} \hat{X}\left(k\right)=\hat{X}\left(k|k-1\right)+\varrho \left[k,\hat{X}\left(k-1\right),{Ƶ}_{q,k}\right] \end{array}$$

where $\hat{X}\left(k|k-1\right)$ and $\hat{X}\left(k\right)$ denote the prediction and estimation of the target state at the *k*th frame, respectively, $\hat{F}$ is the state prediction function, and ϱ represents the gain function that is used to update the target state prediction according to the measurements ${Ƶ}_{q,k}$ for radar *q*.

### 2.3. Pulse Interception and Signal Identification

In a countermeasures environment, radar self-hiding is an essential way to reduce the risk of being jammed, and the key to self-hiding is to improve the LPI performance. There are several indicators that can be adopted to evaluate the LPI performance for radar systems [9,23], such as probability of intercept. In this paper, the number of intercepted pulses is integrated to characterize the LPI performance for the radar system.

The pulses transmitted by the radars will be intercepted by the interceptor with a probability, expressed as [24,25]

(12) $$p_{q,k}^{i}\left(P_{q,k}^{t}\right)=\varsigma_{q,k}^{i}\times \frac{1}{2}\mathrm{erfc}\left(\sqrt{-\ln p_{f}}-\sqrt{\frac{P_{q,k}^{t}G_{q}^{r}G_{I}\lambda_{q}^{2}G_{I}^{P}}{{\left(4\pi R_{q,k}\right)}^{2}k_{b}T_{0}B_{I}F_{I}}+\frac{1}{2}}\right)$$

where erfc(·) is the error function, given by

(13) $$\begin{array}{c} \mathrm{erfc}\left(\varphi \right)=1-\frac{2}{\sqrt{\pi }}\int_{0}^{\varphi }e^{-\zeta^{2}}d\zeta . \end{array}$$

In addition, $\varsigma_{q,k}^{i}$ represents the intercept probability of the dimensions apart from the power dimension, $p_{f}$ is a given false alarm probability of the interceptor, $\lambda_{q}$ is the signal wavelength of radar *q*, $G_{I}$ and $G_{q}^{t}$ are the antenna gain of the interceptor and the radar *q*, respectively. $G_{I}^{P}$ represents the processing gain of the interceptor, $T_{0}$ is the effective noise temperature in Kelvin, $k_{b}$ is the Boltzmann constant, $F_{I}$ and $B_{I}$ are the noise factor and bandwidth of the interceptor, respectively.

**Remark** **2.** *According to* (12)*, the intercept probability $p_{q,k}^{i}\left(P_{q,k}^{t}\right)$ is related to two variables, i.e., the transmit power $P_{q,k}^{t}$ and the radar–target range $R_{q,k}$. In other words, for the same $R_{q,k}$, increasing $P_{q,k}^{t}$ will surge the probability of the pulse being intercepted.*

In the environment without suppressive jamming, radar *q* can transmit, receive, and process $N_{q,k}$ pulses within the *k*th frame for pulse coherent accumulation to improve the signal-to-noise ratio (SNR). Theoretically, the interceptor needs to intercept multiple pulses before the transmit power of the radar can be identified [26]. For the interceptor, there is a confidence threshold, denoted by $\gamma_{t}$, and the interceptor detects the transmit pulses one after another sequentially with the probability of $p_{q,k}^{i}$. If the total number of intercepted radar pulses, denoted by $N_{q,k}^{I}$, meets the confidence threshold $\gamma_{t}$, i.e., $N_{q,k}^{I}=\gamma_{t}$, then the transmitted signal of the radar will be identified. The index of the identified pulse is defined as ${\tilde{N}}_{q,k}$, and the pulse after ${\tilde{N}}_{q,k}$ will be jammed [17]. Since the interception of a radar pulse is a probabilistic event, it is obvious that if the radar signal is identified; then, ${\tilde{N}}_{q,k}⩾\gamma_{t}$ usually holds. Moreover, a large $p_{q,k}^{i}$ means a smaller ${\tilde{N}}_{q,k}$, and if $p_{q,k}^{i}=1$, then ${\tilde{N}}_{q,k}=\gamma_{t}$ holds.

## 3. Problem Modeling

As pointed out in Section 2.2, the higher SINR implies a more efficient tracking performance. Naturally, the power allocation strategy becomes an important factor to improve the SINR and thus tracking accuracy. However, constrained by the presence of suppressive jamming, there is a contradiction for the power allocation problem in the radar network tracking application under a countermeasures environment, which is as follows:

(1) Obviously, increasing the transmit power is an important way to improve the tracking accuracy. However, the excessive transmit power will also increase the probability $p_{q,k}^{i}\left(P_{q,k}^{t}\right)$ in a countermeasures environment, thus raising the risk that the radar will be jammed, negatively affecting the SINR of the radar network and thus decreasing the tracking accuracy;
(2) Reducing the radar transmit power to excessively pursue a lower probability of intercept is also unwise for the radar, which will result in a low SINR, and the strength of the radar will not be fully utilized. For example, the easiest way to achieve the lowest probability of intercept is to radiate no energy into its surveillance area (usually called radio silence), but at the same time, the radar is unable to perform the tracking task (unless it is required to perform a reconnaissance task).

Meanwhile, the target RCS can also affect the SINR, as described in Section 2.1. That is, to obtain a certain SINR, the radar is allowed to transmit less power when a larger value of the target RCS is available, while if the target RCS is smaller, then the radar needs to increase its transmit power appropriately. Obviously, the target RCS can be used to provide significant guidance for radar transmit power adjustment. Next, the relationship between radar transmit power and tracking accuracy, as well as the relationship between target RCS and radar transmit power under equivalent SINR, is presented.

### 3.1. Relationship Between Tracking Accuracy and SINR

After coherent accumulation, the SINR of radar *q* at the *k*th frame can be expressed as

(14) $$\begin{array}{c} \mu_{q,k}\left(P_{q,k}^{t},\beta_{q,k}\right)=\frac{{\left(N_{q,k}\right)}^{2}P_{q,k}^{t}{\left(\beta_{q,k}\right)}^{2}\alpha_{q,k}}{N_{q,k}\sigma_{w}^{2}+\left(N_{q,k}-{\tilde{N}}_{q,k}\right)\sigma_{ja}^{2}}. \end{array}$$

In a countermeasures environment, the trend of tracking accuracy and transmit power can be determined as follows:

• If the radar is not jammed and $\sigma_{ja}^{2}=0$, then the increasing transmit power will improve SINR, where the SINR is equal to the SNR;
• As the transmit power of the radar continues to increase, it will suffer from suppressive jamming, resulting in a sharp decrease in SINR.

**Figure 2:**
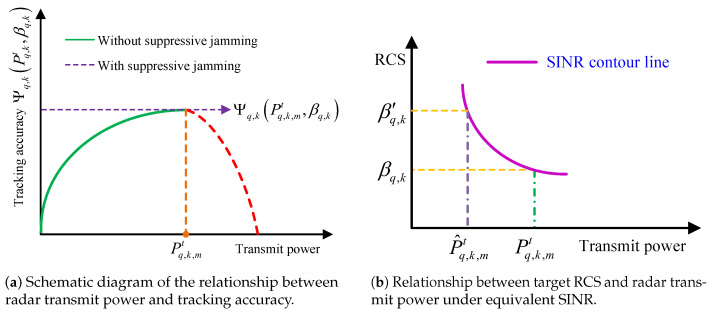
Relationship between radar transmit power, tracking accuracy, and target RCS.

Recalling Section 2.2, the SINR directly determines the measurement covariance matrix, that is, the higher SINR yields a better tracking accuracy [13,18,20]. Following the above analysis, a general relationship between radar tracking accuracy $\Psi_{q,k}\left(P_{q,k}^{t},\beta_{q,k}\right)$ and radar transmit power can be concluded, which is shown in Figure 2a.

More specifically, there should be an optimal transmit power $P_{q,k,m}^{t}$ at the *k*th frame so that radar *q* can achieve the best tracking accuracy $\Psi_{q,k}\left(P_{q,k,m}^{t},\beta_{q,k}\right)$ in a countermeasures environment. Obviously, the power $P_{q,k,m}^{t}$ is related to the probability $p_{q,k}^{i}\left(P_{q,k}^{t}\right)$ and should vary with the radar–target range.

In addition, the diversity of target RCS makes it an important factor to be considered to adjust the radar transmit power. The relationship between target RCS and radar transmit power under equivalent SINR (i.e., equivalent tracking accuracy) can be shown in Figure 2b. Here, assume that the target RCS $\beta_{q,k}^{\prime }>\beta_{q,k}$, combined with (2); theoretically, the radar transmit power is inversely proportional to the target RCS under equivalent SINR. Take the case illustrated in Figure 2b, for example, assume the target RCS is constant, $\beta_{q,k}=\beta_{q,k-1}$, then the range-matched optimal power $P_{q,k,m}^{t}$ can be obtained by the IMP-based power allocation algorithm [17]. If the real value of the target RCS at the *k*th frame is $\beta_{q,k}^{\prime }$, the optimal transmit power $P_{q,k,m}^{t}$ can be adjusted to ${\hat{P}}_{q,k,m}^{t}$ in the direction of becoming smaller. This adjustment step is only related to the variation in the target RCS; in other words, the variation in the RCS can be regarded as the driver of the radar transmit power adjustment.

**Figure 3:**
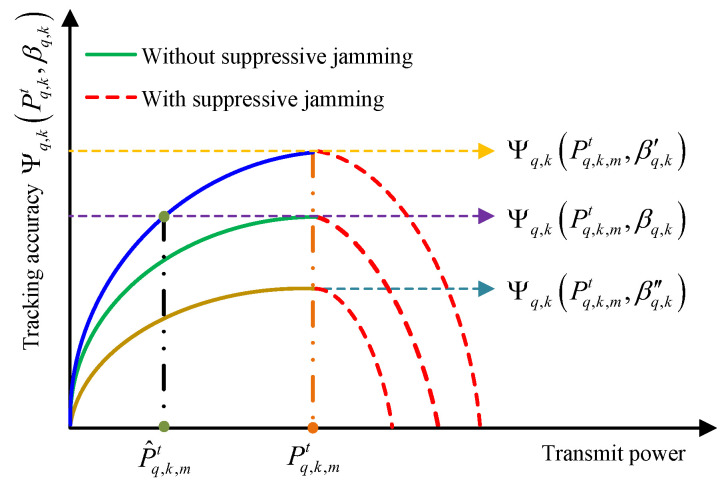
Schematic diagram of the relationship between radar transmit power and tracking accuracy under different RCSs.

Then, the relationship between radar transmit power and tracking accuracy under different RCSs can be obtained and is shown in Figure 3, where $\beta_{q,k}^{\prime }>\beta_{q,k}>\beta_{q,k}^{\prime \prime }$.

It can be seen from Figure 3 that for different target RCSs, the transmit power required for the radar to achieve optimal tracking accuracy is different. Indeed, if the target RCS $\beta_{q,k}$ is taken as the benchmark, the optimal transmit power $P_{q,k,m}^{t}$ can be regarded as the maximum transmit power at which the transmit signal cannot be identified, and the tracking accuracy in this case corresponds to $\Psi_{q,k}\left(P_{q,k,m}^{t},\beta_{q,k}\right)$. (1) When the target RCS is larger, i.e., $\beta_{q,k}^{\prime }>\beta_{q,k}$, the radar tracking accuracy rises gradually with the increase in radar transmit power exceeding $\Psi_{q,k}\left(P_{q,k,m}^{t},\beta_{q,k}\right)$ until the power reaches $P_{q,k,m}^{t}$ and the tracking accuracy achieves the optimal value of $\Psi_{q,k}\left(P_{q,k,m}^{t},\beta_{q,k}^{\prime }\right)$. Meanwhile, the transmit power of the radar in this case can also be adjusted to ${\hat{P}}_{q,k,m}^{t}$ to achieve the equivalent tracking accuracy while saving energy. (2) When the target RCS is smaller, i.e., $\beta_{q,k}>\beta_{q,k}^{\prime \prime }$, even if the radar transmit power reaches $P_{q,k,m}^{t}$, the tracking accuracy can only achieve $\Psi_{q,k}\left(P_{q,k,m}^{t},\beta_{q,k}^{\prime \prime }\right)$. Limited by the smaller target RCS, the radar echo SINR is also smaller, leading to lower tracking accuracy. However, the transmit power of the radar cannot increase any further because the increased transmit power will cause jamming, which leads to a drastic decrease in the tracking accuracy.

Therefore, adaptively allocating the transmit power of the radar according to the variation in the target RCS is crucial for improving the tracking performance in radar network as well as maintaining a good LPI performance. Next, the above problem is modeled and analyzed.

### 3.2. Optimization Model

More generally, it is important to find the minimum allowed radar transmit power that maximizes the tracking accuracy at the current frame. This power ${\hat{P}}_{q,k,m}^{t}$ helps the radar network to obtain the optimal tracking performance on the one hand and minimizes the risk of radar being jammed on the other. Since the interceptor parameters are not available for the radar, ${\hat{P}}_{q,k,m}^{t}$ cannot be obtained directly by the existing optimal algorithms.

Although both the radar transmit power and the target RCS can affect the SINR and tracking accuracy, the causes of the changing tracking accuracy are different. Specifically, they are as follows:

(1) From the perspective of radar transmit power, the SINR will decrease due to the radar being jammed, which in turn leads to a decrease in tracking accuracy. Therein, the intercept probability of the radar pulse is related to the transmit power of the radar and the radar–target range.
(2) In the target RCS perspective, different RCSs will yield different echo power and lead to different SINRs under the same transmit power, which is only related to the target RCS.

This allows us to build the novel algorithm based on the IMP power allocation algorithm (referred to as the IMP algorithm in the following part) presented in our earlier paper [17] (which yields $P_{q,k,m}^{t}$) and combine it with the consideration of the target RCS variation to further yield ${\hat{P}}_{q,k,m}^{t}$. It can be viewed as adjusting $P_{q,k,m}^{t}$ to ${\hat{P}}_{q,k,m}^{t}$ at the *k*th frame. Assume that the target motion is continuous and the target does not move violently during two frames, then $P_{q,k,m}^{t}$ at the *k*th frame can be obtained based on the tracking result of the (k−1)th frame. Combined with the predicted target RCS at the *k*th frame, results can be further optimized, and ${\hat{P}}_{q,k,m}^{t}$ can be obtained at the *k*th frame. Then, the AIMP-based power allocation algorithm for radar network tracking is established, and the detailed steps of the proposed algorithm are described in the Section 4.

## 4. AIMP-Based Power Allocation Algorithm

After the above analysis, it can be seen that the prediction of the target RCS is an important step for adjusting the transmit power allocation strategy. The modeling of target RCS has been partially investigated in [27], where the target RCS is modeled as an ellipsoid model by using the elevation angle and azimuth angle between the radar and the target. In this section, a target motion trajectory-based RCS prediction method is introduced, and on this basis, an AIMP-based power allocation algorithm for the radar network tracking is proposed.

Although the radar electromagnetic frequency and other factors can also affect the target RCS value exhibited to the radar, in this paper, only the effect of the radar observation orientation on the target RCS is focused on, and other factors are omitted.

### 4.1. Radar Incidence Angle

In order to facilitate the description, it is assumed that the target is flight smooth, and the effect of the pitch angle on the target RCS is ignored. Therefore, the radar tracking scenario can be downscaled from a three-dimensional space to a two-dimensional Cartesian coordinate.

**Figure 4:**
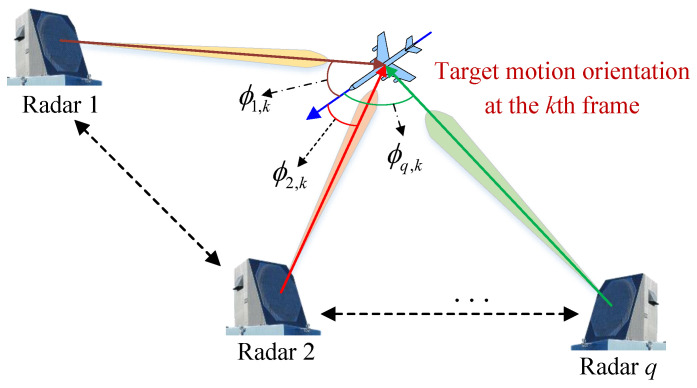
Sketch map of the incidence angle.

When the frequency and polarization mode of the transmit signal are fixed, the target will exhibit different RCS values according to its different sectors observed by the radar, which depends on the radar incidence angle (hereafter referred to as the incidence angle) on the target. The incidence angle is the angle between the direction of radar *q* radiates on the target and the direction of the target motion, denoted as $\phi_{q,k}$. The schematic diagram of the incidence angles is shown in Figure 4.

The incidence angle $\phi_{q,k}$ can be used to indicate the sector of radar *q* radiating on the target at the *k*th frame. Obviously, due to the symmetry of the left and right halves of the target reflective surface and the limitation of the cosine theorem, $\phi_{q,k}\in \left[0,\pi \right]$, and, for example, $\phi_{q,k}=0$ means that radar *q* radiates on the frontal sector of the target at the *k*th frame.

**Figure 5:**
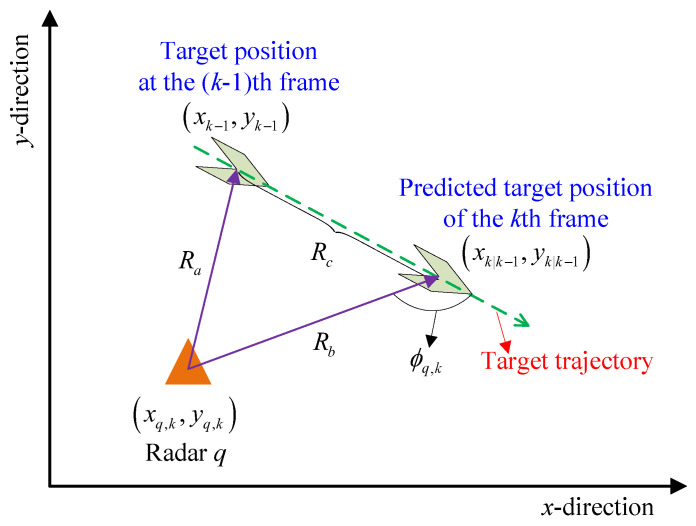
Sketch map of the relative position of the target within two frames.

The incidence angle $\phi_{q,k}$ is related to the trajectory of the target within two successive frames. Assume that the target obeys a uniform linear motion during a short time interval, without loss of generality, the relative position of the target’s motion within two successive frames in a two-dimensional Cartesian coordinate can be given in Figure 5.

According to the cosine theorem, the incidence angle can be expressed as

(15) $$\begin{array}{c} \phi_{q,k}=\pi -\arccos\frac{R_{b}^{2}+R_{c}^{2}-R_{a}^{2}}{2R_{b}R_{c}} \end{array}$$

where $R_{a}$, $R_{b}$, and $R_{c}$ represent the range between radar *q* and the target at the (k−1)th frame, the predicted range between radar *q* and the target at the *k*th frame, and the motion distance of the target within two successive frames, respectively, given by

(16) $$\begin{array}{c} R_{a}=\sqrt{{\left(x_{q}-x_{k-1}\right)}^{2}+{\left(y_{q}-y_{k-1}\right)}^{2}} \end{array}$$

(17) $$\begin{array}{c} R_{b}=\sqrt{{\left(x_{q}-x_{k|k-1}\right)}^{2}+{\left(y_{q}-y_{k|k-1}\right)}^{2}} \end{array}$$

(18) $$\begin{array}{c} R_{c}=\sqrt{{\left(x_{k|k-1}-x_{k-1}\right)}^{2}+{\left(y_{k|k-1}-y_{k-1}\right)}^{2}}. \end{array}$$

By now, the predicted incidence angle at the *k*th frame can be obtained.

### 4.2. Target Motion Trajectory-Based RCS Prediction Method

**Figure 6:**
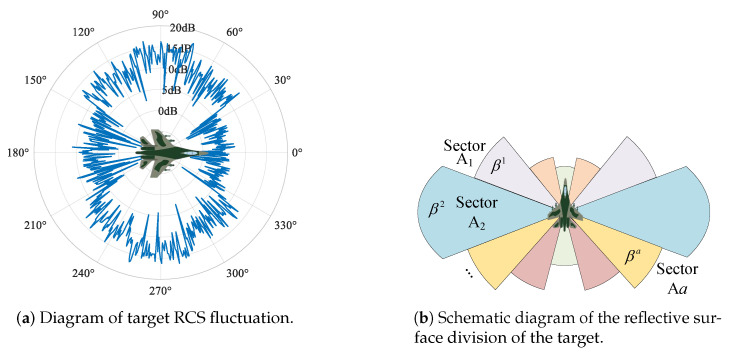
RCS fluctuation and the reflective surface division of the target.

As described in the previous subsection, the target RCS is directly related to the incidence angle, which is affected by the target sector where the radar radiates. In this paper, we assume that the target RCS values obey the Swerling I model; thus, the values of target RCS obey an exponential distribution [14]. To visualize the fluctuation of the target RCS under different incidence angles, the following simulation diagram is shown in Figure 6a.

Divide the reflective surface of the target into several distinct sectors, including the frontal, the lateral, etc., and the RCS corresponding to the *a*th sector $A_{a}$ is assumed to obey the exponential distribution with mean $\beta^{a}$. The schematic diagram of the reflective surface division of the target is shown in Figure 6b.

**Figure 7:**
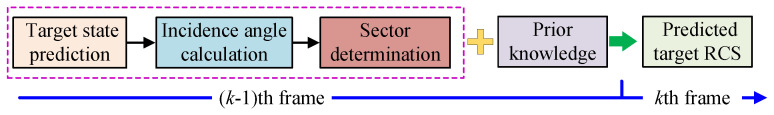
Flowchart of the target motion trajectory-based RCS prediction method.

Thus, at the (k−1)th frame, the target state (i.e., position) can be predicted, and in turn, the radar incidence angle at the *k*th frame can be predicted. Then, determine the target sector that the radar radiates on. Combined with the prior knowledge of RCS value distribution, the predicted target RCS at the *k*th frame can be obtained. The simple flowchart of RCS prediction is shown in Figure 7, and the pseudo-code of the RCS prediction method can be found in Algorithm 1.
**Algorithm 1:** Target motion trajectory-based RCS prediction method.**Input**: The state of the target at the $\left(k-1\right)$th frame and the RCS value distribution.**Output**: The target RCS values at the *k*th frame.1 **Step 1**: Predict the state of the target at the $\left(k-1\right)$th frame;2 **Step 2**: Obtain the ranges $R_{a}$, $R_{b}$ and $R_{c}$ according to (16)–(18);3 **Step 3**: Calculate incidence angle according to (15);4 **Step 4**: Determine the target sector that the radar radiates on based the incidence angle;5 **Step 5**: Obtain the target RCS value at the *k*th frame according to the RCS distribution;

**Remark** **3.** *It is assumed that the corresponding target RCS value of each sector is known as prior knowledge, which can be obtained by the military intelligence or publicly available information.*

### 4.3. AIMP-Based Power Allocation Algorithm

As mentioned in Section 3, in fact, it is difficult to obtain ${\hat{P}}_{q,k,m}^{t}$ directly by using optimization algorithms since the interceptor parameters are unavailable to the radar. The problem can be essentially transformed into an estimate of the ${\hat{P}}_{q,k,m}^{t}$. Borrowing the idea of the interacting multiple model algorithm in target tracking [28,29], the multiple power models can be set up first. Then, according to the **tracking results** and the **target RCS**, the multiple powers and their weights can be updated. Finally, sum the weighted multiple powers to estimate the ${\hat{P}}_{q,k,m}^{t}$.

The steps of the AIMP power allocation algorithm (referred to as the AIMP algorithm in the following part) are given as follows, and for convenience, the subscript *q* is omitted.

***Step 1*** *: Construct multiple power models by processing a different number of echo pulses.*

Assume that the radar transmit power is $P_{k-1}^{t}$ at the (k−1)th frame, and there are *J* echo queues containing different pulses (*j*-th echo pulse queue has $N_{j}$ pulses) processed at the (k−1)th frame to achieve *J* transmitting power values, denoted by $P_{k-1}$ = $\left[P_{k-1,1},P_{k-1,2},\ldots ,P_{k-1,J}\right]$, where

(19) $$\begin{array}{c} P_{k-1,j}=\frac{N_{j}}{N_{J}}P_{k-1}^{t},\left(j=1,2,\ldots ,J\right). \end{array}$$

The multiple power weights are denoted as $\eta_{k-1}$. For simplicity, the detailed calculation of $\eta_{k-1}$ can be found in reference [17].

***Step 2*** *: Update the multiple power weights.*

It should be noted that if the echo queues contain a jammed pulse, the target may not be detected by the radar. After processing *J* echo queues, *J* filtering results can be obtained, and those results can be utilized as the feedback to update the multiple power weights for the *k*th frame, $\eta_{k}=\left[\eta_{1,k},\eta_{2,k},\ldots ,\eta_{J,k}\right]$. More details of *Step 1* and *2* can be found in our previous paper [17].

***Step 3*** *: Predict the target RCS at the kth frame.*

In *Step 3*, the target RCS for the *k*th frame can be predicted by Algorithm 1.

***Step 4*** *: Update the multiple powers.*

Similarly, the multiple powers based on whether the radar is jammed can be updated in the current frame, while the change in target RCS can guide the adjustment of multiple power.

**Figure 8:**
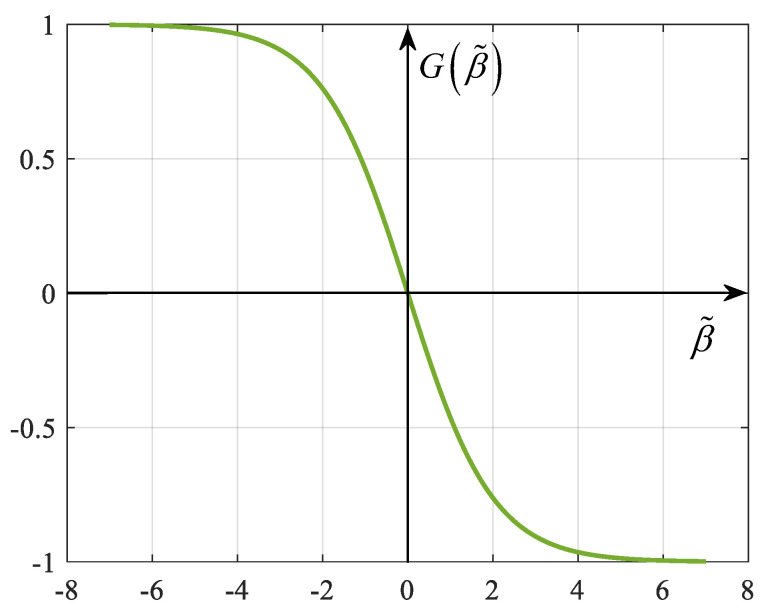
Graph of the improved Sigmoid function.

*Case 1:* The radar is not jammed. The main idea of power adjustment was analyzed previously; that is, when the target RCS predicted at the *k*th frame increases corresponding to that of the (k−1)th frame, the allocated power $P_{k,m}^{t}$ should be appropriately decreased, and vice versa. It means that the change in the target RCS dominates the power adjustment. Let Δβ denote the change in target RCS, given by

(20) $$\begin{array}{c} \Delta \beta =\frac{{\left(\beta_{k}\right)}^{2}}{{\left(\beta_{k-1}\right)}^{2}}. \end{array}$$

where $\beta_{k}$ ($\beta_{k-1}$) is the RCS value at the *k*th((k−1)th) frame. To further characterize the driving effect of RCS change on radar transmit power adjustment, let $G\left(\tilde{\beta }\right)$ denote the adjustment factor of $P_{k,m}^{t}$. According to the improved Sigmoid function, a correspondence can be constructed as follows:

(21) $$\begin{array}{c} G\left(\tilde{\beta }\right)=a\left(b-\frac{1}{1+e^{\tilde{\beta }+\varepsilon }}\right) \end{array}$$

where parameters *a*, *b*, and ε determine the maximum (minimum) value, location of the center, and the zero coordinates of the function, respectively. In order to avoid a drastic increase or decrease in power, some restrictions are set on this correspondence, i.e., make a=2, b=0.5 and ε=0. Then, the graph of the function is shown in Figure 8.

In addition, $\tilde{\beta }$ is the function of Δβ, given by

(22) $$\begin{array}{c} \tilde{\beta }=\left\{\begin{array}{cc} \Delta \beta & \Delta \beta >1\\ 0 & \Delta \beta =1\\ -\frac{1}{\Delta \beta } & \Delta \beta <1 \end{array}\right. \end{array}$$

Then, the multiple powers can be updated as

(23) $$\begin{array}{c} {\tilde{P}}_{k}=\left[1+G\left(\tilde{\beta }\right)\right]P_{k-1}+\Delta P_{k-1} \end{array}$$

In particular, when Δβ=1 holds, $\tilde{\beta }=0$ holds, which implies that the RCS values are approximately the same in the two successive frames, and the driving power adjustment by the RCS change is weakened. Thus, the AIMP-based power allocation algorithm degenerates into an IMP algorithm, which is proposed in our prior work [17].

*Case 2:* The radar is jammed. In this case, multiple powers can be updated first to ${\tilde{P}}_{k-1}$ based on Equation (30) in [17] and then further updated according to (23).

***Step 5**: Obtain the power ${\hat{P}}_{k,m}^{t}$.* Using the updated multiple power ${\tilde{P}}_{k}$ and weights $\eta_{k}$, we can thereafter obtain the optimal power ${\hat{P}}_{k,m}^{t}$ at the *k*th frame,

(24) $$\begin{array}{c} {\hat{P}}_{k,m}^{t}={\tilde{P}}_{k}{\left(\eta_{k}\right)}^{T} \end{array}$$

In addition, at the (k−1)th frame, the information fusion techniques are employed after the target state $\hat{X}\left(k-1\right)$ is estimated by processing the echo signals.

**Figure 9:**
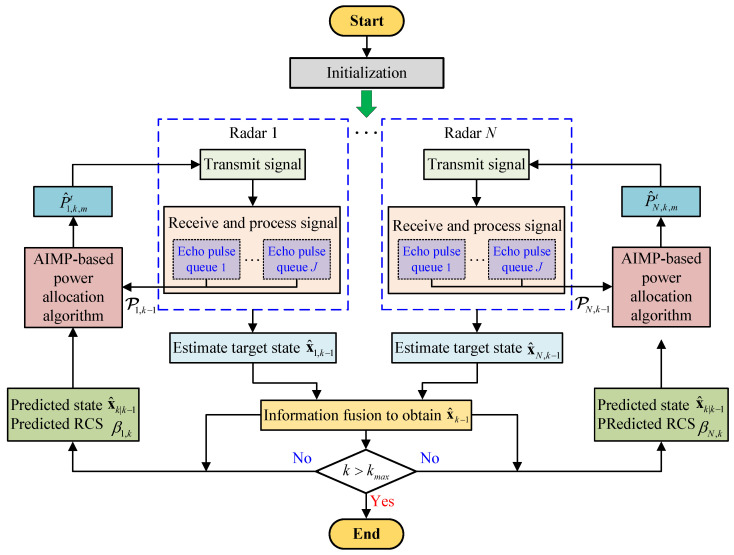
Overall flowchart of radar network target tracking signal processing with the AIMP algorithm.

The overall flowchart of the radar network tracking signal processing with AIMP-based power allocation algorithm is shown in Figure 9. After initialization, at the $\left(k-1\right)$th frame, radar *q* transmits $N_{q,k}$ pulses with power ${\hat{P}}_{q,k-1,m}^{t}$ to detect the target. Then, after reflection from the target, radar *q* receives and processes *J* radar echo queues associated with its own transmit signal. Subsequently, after the necessary filtering steps, the radar *q* can obtain an estimation of the target state based on its own measurement. Next, the estimation of the target state obtained from each radar in the radar network is fused, and the fused target state is used for target RCS and state prediction. Finally, the tracking process at the (k−1)th frame is finished. Meanwhile, the AIMP-based power allocation algorithm is utilized to obtain the power ${\hat{P}}_{q,k,m}^{t}$ of radar *q* at the *k*th frame and start the tracking process for the *k*th frame.

## 5. Simulation Results

In this section, some simulation results are given to demonstrate the effectiveness and advantages of the proposed AIMP power allocation algorithm (referred to as the AIMP algorithm in the following part) with a typical application scenario. Specifically, the proposed algorithm is compared with the IMP power allocation algorithm (referred to as the IMP algorithm in the following part) [17] and the fixed power allocation algorithm with the maximum power $P_{max}=20$ kW. The results given below are based on $M_{c}=300$ independent Monte Carlo trials.

### 5.1. Simulation Scenario

**Figure 10:**
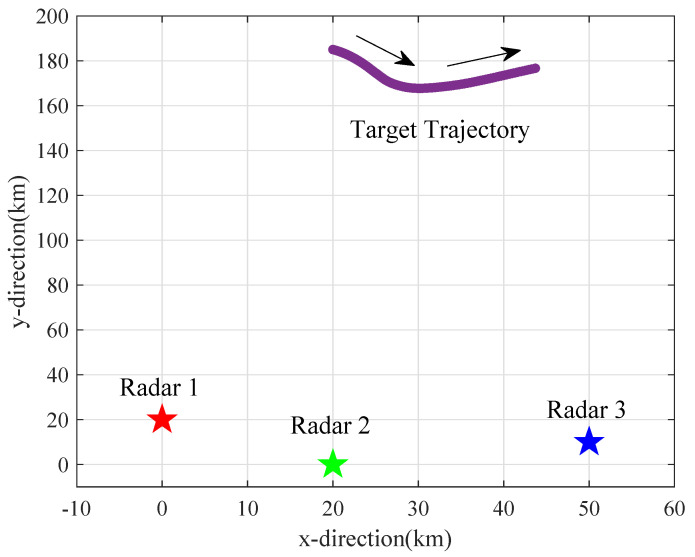
Schematic diagram of simulation scene. The arrows indicate the direction of the target’s movement.

The simulation scenario is shown in Figure 10. There are three radars in the radar network monitoring a surveillance area and tracking a target. Therein, the pentagrams represent positions of each radar, assigned as $\left(0,20\right)$ km, $\left(20,0\right)$ km and $\left(50,10\right)$ km, respectively. The purple curve represents the target trajectory, and the direction of the target is marked by the black arrow. The total number of frames $k_{max}$ is 100.

Since the initial two frames need to be used to initialize the parameters of radar network tracking, the following simulation results are shown from the 3rd frame, which does influence the validity of the results.

### 5.2. Simulation Parameters

The parameters of this simulation consist of four parts:

*Part 1: Parameters about the radars.* Assume that all the radars in the network are homogeneous. The parameters of the *q*th radar are as follows. The number of transmit pulses is fixed at each frame $N_{q,k}=16$, the signal effective bandwidth and wavelength are $B_{q}=5\;\mathrm{MHz}$ and $\lambda_{q}=0.05\;m$, respectively. The gain of radar antenna and aperture are $G_{q}^{r}=36\;\mathrm{dB}$ and $A_{q}=2.25$ m^2^, respectively.

*Part 2: Parameters about the interceptor.* The receiver antenna gain is $G_{I}=3\;\mathrm{dB}$, the process gain is $G_{I}^{P}=3\;\mathrm{dB}$, the bandwidth is $B_{I}=20\;\mathrm{GHz}$, the false alarm probability and noise factor are $p_{I,fa}=10^{-6}$ and $F_{I}=12\;\mathrm{dB}$, respectively.

*Part 3: Parameters of the target RCS.* In this paper, the reflective surface of the target is axisymmetrically divided into five distinct sectors. The extent of each sector (in terms of incidence angle $\phi_{q,k}$) and its corresponding average RCS value are given in Table 1, wherein the RCS of the *a*th sector obeys the exponential distribution with mean $\beta^{a}$. The mean value of RCS in the comparison algorithm is 5 m^2^.

*Part 4: Parameters of the proposed algorithm.* The initial multiple power is ${\tilde{P}}_{0}=\left[8000,10,000,15,000\right]\;W$, and the initial weight vector is $\eta_{0}=\left[1/3,1/3,1/3\right]$. The probability $\varsigma_{q,k}^{i}$ is a constant value of 0.7. Additional parameters can be found in [17].

### 5.3. Target Range and RCS Values Exhibited to Different Radars

**Figure 11:**
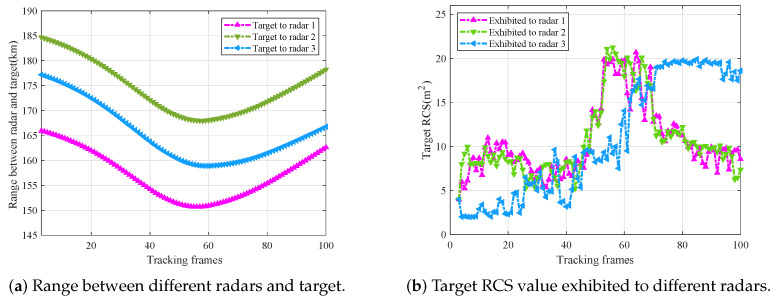
Target range and RCS values to different radars.

In this paper, the effects of the variable radar–target range as well as the target RCS on radar power allocation are focused. The variable range between radars and the target is shown in Figure 11a, and the values of target RCS exhibited to different radars are shown in Figure 11b.

Combined with the scene graph shown in Figure 10, taking Radar 1 as an example, from the beginning of tracking to about the 50th frame, the incidence angle of Radar 1 is in the interval of $\left[30^{\circ },60^{\circ }\right)$, and the average value of the target RCS is around 8 m^2^ during this period. As the target turns and maneuvers, from about the 50th frame to the 100th frame, the target is radiated from the wing sector to the tail sector, and the incidence angle changes from the interval of $\left[60^{\circ },120^{\circ }\right)$ to $\left[120^{\circ },150^{\circ }\right)$, resulting in the average target RCS value changing from 20 m^2^ to about 10 m^2^ at this stage. The target RCS values exhibited to Radar 2 and 3 can be analyzed in the same way, and the turning points are all around the 50th frame.

### 5.4. Power Allocation Results and Tracking Performance Comparison

**Figure 12:**
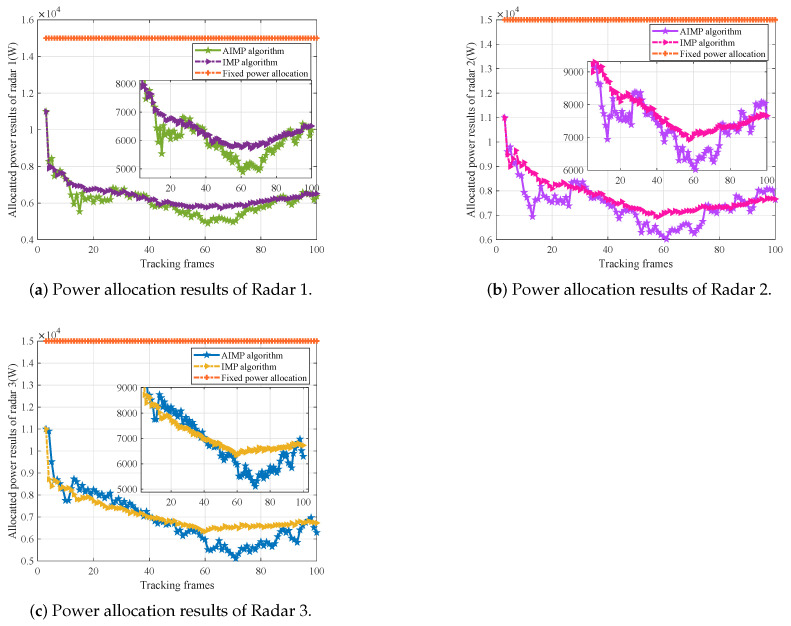
Power allocation results of each radar with three power allocation methods.

The power allocation results of each radar with the proposed AIMP algorithm and the IMP algorithm, as well as the fixed power allocation algorithm, are shown in Figure 12a, Figure 12b, and Figure 12c, respectively.

Combined with the radar–target range in Figure 11a and target RCS variation in Figure 11b, the analysis of the results is given next.

As shown in Figure 12a–c, when target RCS tends to increase (i.e., from about 45th frame to 55th frame for radar 1, 2, and from about the 60th frame to the 70th frame for radar 3), based on the AIMP algorithm, the parameters satisfy Δβ>1, $\tilde{\beta }=\Delta \beta$, $G\left(\tilde{\beta }\right)<0$, so the allocated transmit power is reduced in this period. When the target RCS tends to decrease (i.e., from about the 65th frame to the 75th frame for radar 1, 2), based on the AIMP algorithm, the parameters satisfy Δβ<1, $\tilde{\beta }=-\frac{1}{\Delta \beta }$, $G\left(\tilde{\beta }\right)>0$, so the allocated transmit power is raised. When the target RCS remains essentially constant (e.g., from about the 10th frame to the 40th frame for radar 1, 2, 3), based on the AIMP algorithm, the parameters are Δβ=1, $\tilde{\beta }=0$, $G\left(\tilde{\beta }\right)=0$, so the radar transmit power will remain stable (with a small increase) as well.

Overall, the AIMP power allocation algorithm, which considers both the radar–target range and the target RCS, can allocate the transmit power more optimally and conservatively compared with the IMP algorithm, which only considers the radar–target range, wasting less power.

**Figure 13:**
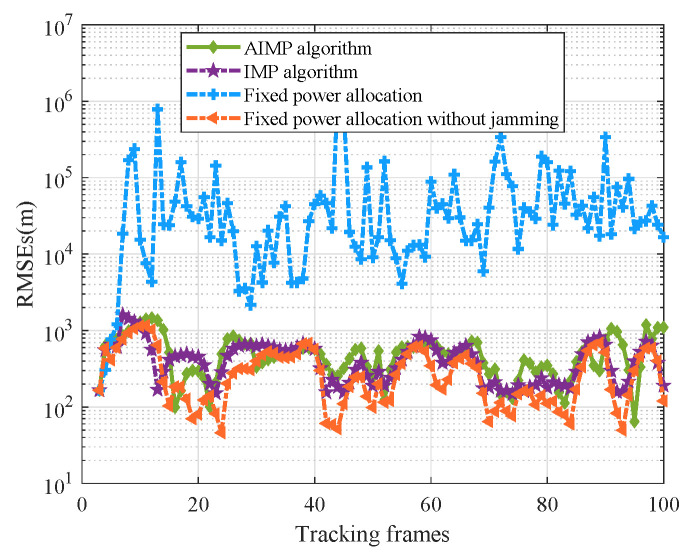
Tracking RMSEs of the radar network with different power allocation methods.

Correspondingly, the tracking performance of the radar network using the proposed power allocation algorithm is also compared with the existing algorithms, and the tracking effectiveness is characterized by the root mean square error (RMSE)

(25) $$\begin{array}{c} {\mathrm{RMSE}}_{k}=\sqrt{\frac{1}{M_{c}}\sum_{s=1}^{M_{c}}\left[{\left(x_{k}-{\hat{x}}_{k,s}\right)}^{2}+{\left(y_{k}-{\hat{y}}_{k,s}\right)}^{2}\right]} \end{array}$$

where $M_{c}$ is Monte Carlo trials number and $\left({\hat{x}}_{k,s},{\hat{y}}_{k,s}\right)$ is the target state estimate of the *s*th trail. In each frame, the information fusion techniques are applied to correlate the tracking data from each radar in the network to estimate the target state [2]. In order to verify the effectiveness of the proposed power allocation algorithm in target tracking, the tracking RMSE based on the proposed AIMP algorithm, the IMP algorithm, and the fixed power allocation algorithm, as well as the fixed power allocation algorithm without suppressive jamming, are compared and shown in Figure 13. Here, the value of the fixed power is set to be $P_{max}=20\;$ kW.

As can be seen in Figure 13, with the fixed power allocation algorithm, the transmit power of each radar in the radar network cannot be adjusted, which in turn leads to radar pulses being intercepted and signal being identified in case of short radar–target range. Then, the intercepted radar will be jammed. Thus, the radar is unable to track the target normally, and the tracking RMSE increases. However, the tracking performance of the AIMP algorithm and the IMP algorithm is basically approximate to that of the fixed power allocation without jamming, consuming less power. Looking back at the power allocation results in Figure 12, the average transmit power of radars with the AIMP and IMP algorithms is given in Table 2.

Combining Table 2 with Figure 13, it can clearly be seen in these results that the radar network will consume less power with the AIMP algorithm to achieve equivalent tracking effectiveness compared with the IMP algorithm.

### 5.5. LPI Performance Comparison

**Figure 14:**
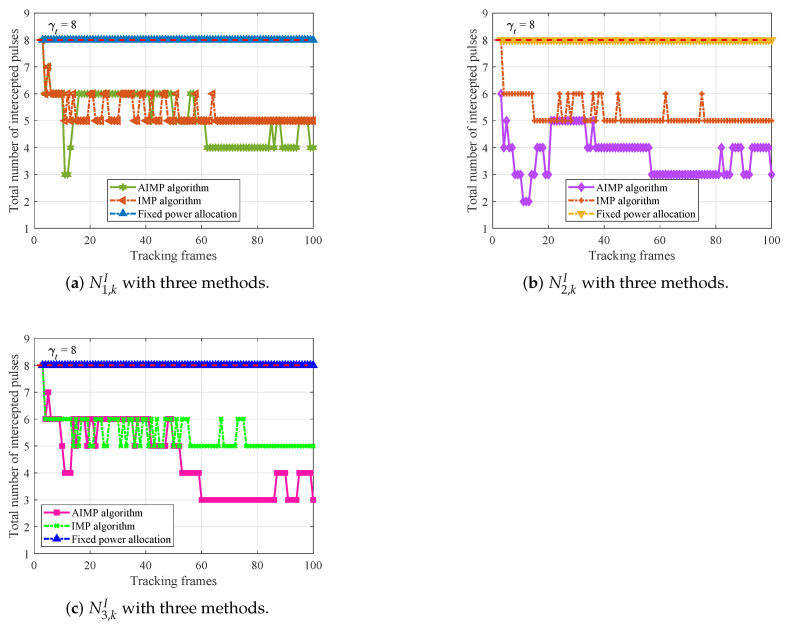
LPI performance comparison of each radar with three power allocation methods.

As mentioned in Section 2.3, once the interceptor has intercepted enough $\gamma_{t}$ radar pulses, the radar will be jammed. That is, $N_{q,k}^{I}<\gamma_{t}$ should be held for radar *q* to achieve good LPI performance. Therefore, next, the intercepted pulse number of the radars in the network is compared between the AIMP algorithm, the IMP algorithm, and the fixed power allocation algorithm to demonstrate that the radar network can achieve good LPI performance with the proposed AIMP algorithm. Set the confidence threshold to $\gamma_{t}=8$. After Monte Carlo simulations, it can be seen from Figure 14a–c that the intercepted pulse number $N_{q,k}^{I}$ in each frame of the radars based on AIMP algorithm and IMP algorithm is always less than $\gamma_{t}$, which leads to that the radar signal cannot be identified, i.e., the radar cannot be jammed.

Obviously, $N_{q,k}^{I}$ is directly related to the transmit power, and according to the analysis in the previous subsection, the AIMP algorithm can achieve an equivalent tracking accuracy while consuming less power, obtaining better anti-interception capability of the radar network or LPI performance. Furthermore, the superior LPI performance enables the radar network to track targets more robustly in a countermeasures environment.

On the contrary, if the radar transmits a signal at a fixed power $P_{max}$, there is an increased risk that the radar pulses will be intercepted and the signal will be identified; thus, the radar faces the risk of being jammed, which leads to higher tracking RMSE. Take Radar 1, for example, based on the fixed power allocation algorithm, the radars are suppressed for almost the entire tracking process due to the close proximity between Radar 1 and the target. In summary, by jointly considering the radar–target range and the target RCS, with the proposed AIMP algorithm, the LPI performance of the radar network can be further improved.

### 5.6. Limitations of the AIMP-Based Power Allocation Algorithm

Although the effectiveness of the proposed algorithm is proved through simulation results, it still faces some limitations.

First, there is a large uncertainty in the prediction of the target RCS in practical applications. Targets tend to execute turns, rolls, and other maneuvers in motion, which raises a higher challenge for the prediction of target RCS. Therefore, the target RCS prediction method proposed in this paper is relatively fundamental, but it provides ideas for radar power allocation, i.e., obtaining the target RCS will be conducive to the adjustment of radar transmit power.

Moreover, the proposed algorithm does not emphasize the cooperation between radar nodes. In fact, different radar nodes observe different values of target, and thus, depending on the cooperation way of the radar network, the radar transmit power can be further optimized.

## 6. Conclusions

In this paper, the problem of power allocation for radar network tracking in a countermeasures environment is investigated. The effect of suppressive jamming on the radar echo pulse, as well as the spatial diversity of target RCS, is fully considered, and an AIMP-based power allocation algorithm for radar network tracking is proposed. Numerical simulations demonstrate that, by considering the variation in target RCS, the radar network can achieve the equivalent tracking accuracy with the proposed AIMP power allocation algorithm but use less power, while maintaining anti-jamming capability and efficient LPI performance. Obviously, the power allocation algorithm will be more efficient, along with a more accurate RCS prediction. In addition, the cooperation between radars in the network is not adequately considered in this paper, which will be researched further in the subsequent study.

## Figures and Tables

**Table 1:** The extent of each sector and its corresponding average RCS value.

Extent of $\phi_{q,k}$ (in Degrees)	Average RCS Value
$\phi_{q,k}\in \left[0,30^{\circ }\right)$	$\beta^{1}=2$ m^2^
$\phi_{q,k}\in \left[30^{\circ },60^{\circ }\right)$	$\beta^{2}=8$ m^2^
$\phi_{q,k}\in \left[60^{\circ },120^{\circ }\right)$	$\beta^{3}=20$ m^2^
$\phi_{q,k}\in \left[120^{\circ },150^{\circ }\right)$	$\beta^{4}=10$ m^2^
other	$\beta^{5}=4$ m^2^

**Table 2:** Average transmit power of radars with AIMP and IMP algorithms.

		Average Transmit Power		
	Radar	Radar 1	Radar 2	Radar 3
Algorithm				
AIMP-based algorithm		6100.2 W	7438.9 W	6869.7 W
IMP-based algorithm		6391.0 W	7772.3 W	7087.5 W

## Data Availability

Data are contained within this article. All data in this paper are generated by simulation, and the details are presented in Section 5.
